# Age- and sex-dependent increase in self-harm among adolescents with mental health problems in East China during COVID-19 related society-wide isolation

**DOI:** 10.3389/fpubh.2023.1129123

**Published:** 2023-03-09

**Authors:** Wenjing Liu, Zhishan Hu, Zhen Liu, Fang Zhang, Yue Ding, Ying Shui, Zhi Yang, Wenhong Cheng

**Affiliations:** ^1^Department of Child and Adolescent Psychiatry, Shanghai Mental Health Center, Shanghai Jiao Tong University School of Medicine, Shanghai, China; ^2^Laboratory of Psychological Heath and Imaging, Shanghai Mental Health Center, Shanghai Jiao Tong University School of Medicine, Shanghai, China; ^3^Institute of Psychological and Behavioral Science, Shanghai Jiao Tong University, Shanghai, China; ^4^Department of Psychological Medicine, Shanghai General Hospital, Shanghai Jiao Tong University School of Medicine, Shanghai, China

**Keywords:** self-harm, COVID-19, adolescent, society-wide isolation, emotional disorder

## Abstract

**Objective:**

The COVID-19 pandemic has raised concerns about child and adolescent mental health issues, such as self-harm. The impact of society-wide isolation on self-harming behaviors among adolescents in China is unclear. In addition, adolescents of different ages and sexes have varying abilities to cope with environmental changes. However, these differences are rarely considered in self-harm studies. We aimed to characterize the age- and sex-dependent effects of COVID-19-related society-wide isolation on self-harm among adolescents in East China.

**Methods:**

We collected 63,877 medical records of children and adolescents aged 8–18 who had an initial visit to Shanghai Mental Health Center in China between 2017 and 2021 and charted annual self-harm rates for each age and sex. Using interrupted time series analysis, we modeled global and seasonal trends and the effect of COVID-19-related society-wide isolation on self-harm rates.

**Results:**

Females aged 10–17 and males aged 13–16 exhibited significantly increasing trends in self-harm rate (*p*_fdr_ < 0.05) in the past 5 years. Eleven-year-old females in 2020 showed a self-harm rate (37.30%) that exceeded the peak among all ages in 2019 (age 13, 36.38%). The COVID-19-related society-wide isolation elevated self-harm rates in female patients aged 12 [RR 1.45 (95% CI 1.19–1.77); *p*_fdr_ = 0.0031] and 13 years [RR 1.33 (95% CI 1.15–1.5); *p*_fdr_ = 0.0031], while males were less affected. Further, females with emotional disorders dominated the increased self-harm rates.

**Conclusion:**

Society-wide isolation has had a significant impact on early adolescent females in East China, especially for those with emotional disturbances, and has brought forward the peak in adolescent self-harm rates. This study calls for attention to the risk of self-harm in early adolescents.

## 1. Introduction

Self-harm among adolescents has rapidly increased over the last decade (1–3). Adolescents with self-harm behaviors are 30 times more at risk for suicide than those without (4) and typically consume more medical resources (5). Mental health problems are remarkable risk factors for self-harm in children and adolescents. Among children and adolescents with major depressive disorder, the prevalence of self-harm is 55.2–64.1% (6, 7). Self-harm is also commonly comorbid with autism spectrum disorders and eating disorder in children and adolescents (8, 9). Previous studies have also associated self-harm with anxiety and depression symptoms (10–12).

The COVID-19 pandemic and related prevention measures have induced substantial changes in the social environment that have affected everyone's life and mental health (13–15). However, while there is a consensus that children and adolescents are vulnerable to social environment change (16), the impact of the COVID-19 pandemic on self-harm in children and adolescents is unclear. Previous studies have reported inconsistent findings regarding the impact of the COVID-19 pandemic on self-harm among children and adolescents worldwide (17–21). In particular, cultural, racial, and sex differences significantly affect the incidence of self-harm and the impact of the pandemic.

The social environment changes accompanying the nationwide home-study measures in China, which began in March 2020, may increase adolescents' stress and worsen their mental health problems. The term “society-wide isolation” in this study represents the combined effect of COVID-19 prevention measures characterized by society-wide isolation. The social isolation, home-study, and other preventive measures have severely impacted adolescents' emotional state and social activity levels, especially for those with psychiatric disorders (22–25). Studies have found significantly increased levels of anxiety and depression in adolescents following COVID-19 (26–28), which is an essential risk factor of self-harm. Thus, environmental changes associated with COVID-19 are expected to exacerbate self-harm among Chinese adolescents with mental health problems. However, studies in China are rare.

Furthermore, most studies on the effects of COVID-19 on self-harm treat the children and adolescent population as a whole (20, 21, 29). However, as a transitional stage from childhood to adulthood, the social needs of adolescents change rapidly with age, such that adolescents of different ages respond differently to social and environmental stressors (30). Thus, age and sex should be comprehensively considered in understanding the vulnerability of self-harm under the major environmental changes.

Here, we aim to determine the age- and sex-specific effects of COVID-19-related prevention measures, with the primary form of society-wide isolation, on self-harm among children and adolescents in East China. Using medical records of children and adolescents aged 8–18 years (*n* = 60,870), we charted year-to-year changes in the prevalence of self-harm of each age and sex. The effects of society-wide isolation on self-harm detection rate was disentangled from global temporal trends and seasonal variations. The result presents a fine-grained picture of recent trends in self-harm in children and adolescents with mental health problems and the extent to which they are influenced by COVID-19 in China.

## 2. Method

### 2.1. Data source

Retrospective data were obtained from electronic medical records from Shanghai Mental Health Center (SMHC), China, from January 2017 to September 2021. In total, 63,877 records of the initial visits of child and adolescent aged 8–18 were acquired. Three thousand and seven records were excluded due to missing critical information (main complaint, history of present illness, psychiatric interview, and information to confirm age and sex), remaining 60,780 records (female = 34,137, male = 26,733, Table 1). The majority of these patients (85.7%) resided in East China, including Shanghai, Shandong, Jiangsu, Anhui, Zhejiang, Jiangxi, and Fujian provinces. The acquisition and analysis of the data was approved by the Institutional Review Board at SMHC.

**Table 1:** Age and sex distributions of the sample.

**Age**	***N***	**Male (%)**	**Female (%)**	**χ^2^**	***p*_*fdr*_**
8	2,030	543 (26.7)	543 (73.3)	438.98	<0.0001
9	1,985	583 (29.4)	583 (70.6)	337.91	<0.0001
10	1,816	607 (33.4)	607 (66.6)	199.56	<0.0001
11	2,553	1,091 (42.7)	1,091 (57.3)	53.91	<0.0001
12	4,452	2,476 (55.6)	2,476 (44.4)	56.15	<0.0001
13	6,638	4,204 (63.3)	4,204 (36.7)	471.96	<0.0001
14	8,328	5,186 (62.3)	5,186 (37.7)	501.67	<0.0001
15	8,727	5,238 (60)	5,238 (40)	350.52	<0.0001
16	10,155	6,029 (59.4)	6,029 (40.6)	356.61	<0.0001
17	9,556	5,525 (57.8)	5,525 (42.2)	233.57	<0.0001
18	4,630	2,655 (57.3)	2,655 (42.7)	99.87	<0.0001

### 2.2. Measurements and clinical coding

The text in the main complaint, history of present illness, and psychiatric interview were pooled to generate a term dictionary in which we searched for terms related to self-harm without suicidal intent (31, 32). A portion of the search terms were extracted from the Chinese version of the Ottawa Self-Injury Scale (33), and additional terms were selected from the term dictionary, which indicate self-harm or suicide attempt (Supplementary Table S1 lists all search terms). Records matching at least one of the search terms were identified as representing self-harm behavior. Terms referred to in a negative way, such as “no self-harm”, were not considered self-harm terms.

### 2.3. Data analysis

We calculated self-harm detection rates for each sex and age group in each year, as well as annual changes in self-harm rates, i.e., rates that differed from 1 year to the next. Annual changes across years were compared using a bootstrap approach. We first resampled individuals from each sex and age group using the bootstrap method (sampling by replacement while keeping the sample size constant). Then, based on the resampling, annual changes in self-harm rates were recalculated. This procedure was repeated 1,000 times to obtain the sampling distribution of annual changes in self-harm rates for each year since 2018. Finally, we compared the annual change for each year since 2019 with the sampling distribution from previous years. Exceeding the 95th percentiles (i.e., *p* < 0.05) of all previous years' sampling distributions of annual changes was considered a significant change in self-harm rates.

To better quantify the impact of COVID-19-related society-wide isolation, which is marked by the implementation of home-study in most cities in China from March 2020, we further examined monthly self-harm rates. We used interrupted time series (ITS) analysis to disentangle the effects of COVID-19-related society-wide isolation from the global temporal and seasonal trends in self-harm rates (34, 35). The “interruption” here refers to March 2020, from which time point the home-study begins. This model fits monthly self-harm incidence data for each age and sex group. The data for September 2021 were removed from further analysis because we only had data for the first 10 days of this month. The ITS model can be formulated as:

$$\begin{array}{lll} \log\left(\text{nHarm}\right) & = & \log\left(\text{nCount}\right)+\text{COVID}\\ + & \text{Slope}+\text{harmonic}\left(\text{Month},2,12\right)+\text{Global} \end{array}$$

This model assumes that the count of patients with self-harm behavior (nHarm) has a Poisson distribution, and we used a quasi-Poisson distribution to deal with the overdispersion problem (36). Specifically, the “nHarm” denotes the monthly count of patients who engaged in self-harm behavior, and the “COVID” denotes whether the recorded month is before (January 2017 to Feb 2020) or after the declaration of national-wide home study (March 2020 to August 2021). The COVID-19 control measures quickly reached to a peak and were gradually eased till September 2021, though not removed. The “Slope” encodes the elapsed time since the implementation of national-wide home-study, which captures the slope change caused by the society-wide isolation and the gradual ease of the control measures. We used the “harmonic” terms (two pairs of sine and cosine functions) to model the potential influence of the seasonality (37). In addition, monthly patient counts (nCount) were modeled as an offset variable in order to transform the counts of self-harm incidents back to rates. The “Global” is the elapsed time from the first day of the medical records we analyzed, and it captures the global change in self-harming behavior. All the analyses were performed using R (Version 4.1.2).

With this model, we examined whether there was an overall trend of increasing self-harm rates over time and at which age this trend would occur. Further, we investigated the effect of COVID-19-related society-wide isolation on self-harm rates and at which age groups children and adolescents were most affected.

We further hypothesized that emotional disorders are an important contributing factor to the increased self-harm rate associated with COVID-19. We categorized patients into emotional disorders and non-emotional disorders by clinical diagnosis, with depressive and bipolar disorder, anxiety disorder, post-traumatic stress disorder, obsessive compulsive disorder and childhood emotional disorder classified as emotional disorders. The rest, including developmental disorders, schizophrenia, and other behavioral problems, were categorized as non-emotional disorders.

## 3. Results

**Figure 1:**
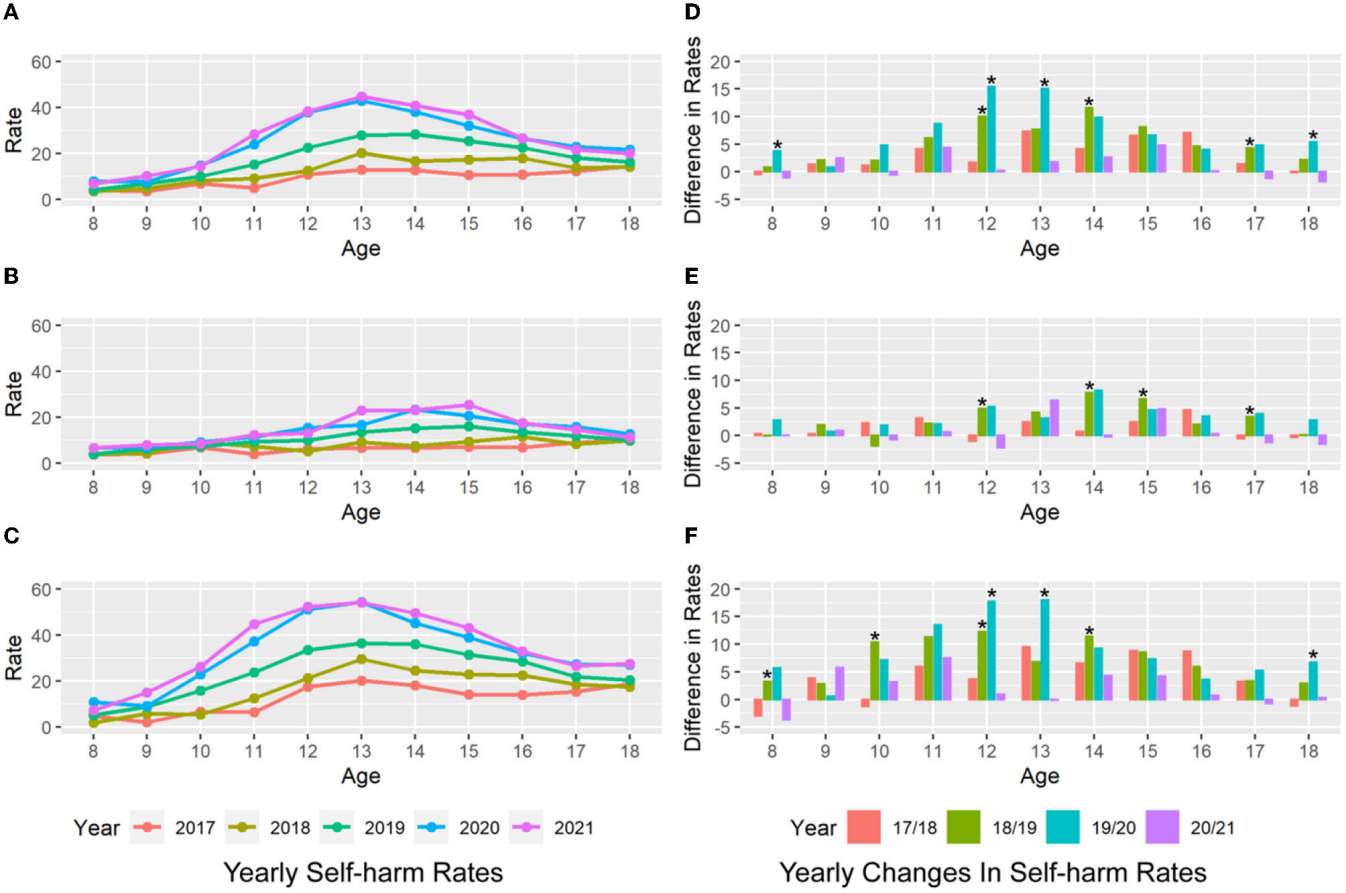
Self-harm rates at different ages. The left panel shows the annual self-harm rates for the entire sample **(A)** of male **(B)** and female patients **(C)** at different ages. The colors indicate the different years of data. Females show an increasing trend in self-harm rates starting at age 10. The right panels show the annual change in self-harm rates for the full sample **(D)**, male **(E)**, and female patients **(F)** at different ages. Females aged 12–13 years show a significantly greater change in self-harm rates between 2019 and 2020 than in previous years (“^*^” represents significance in bootstrapping).

Between 2017 and 2021, the self-harm rates among children patients aged 10–17 increased remarkably over time (Figure 1A). This upward trend started at age 12 among males (Figure 1B) and as early as 10 among females (Figure 1C). In addition, comparing before and after the implementation of COVID-19 prevention and control measures, i.e., from 2019 to 2020, there was a significant jump (*p* < 0.05) in self-harm rates among children aged 12–13 years (Figure 1D). The increase in self-harm rates in these two age groups was 15.39 and 15.02%, respectively, driven mainly by the self-harm rate in female patients (Figure 1F, 17.76 and 17.95%). In contrast, for male children, no significant increase was observed for all age groups from 2019 to 2020 (Figure 1E).

Notably, the self-harm rate for 11-year-old females increased alarmingly from 23.84% in 2019 to 37.30% in 2020 and 44.78% in 2021 (Figure 1C). Following the COVID-19 outbreak, the annual self-harm rate for 11-year-old females in 2020 (37.30%) already exceeded the peak self-harm rate among all ages in 2019 (occurring at age 13, 36.38%). This phenomenon represents a younger trend in self-harm incidents.

**Figure 2:**
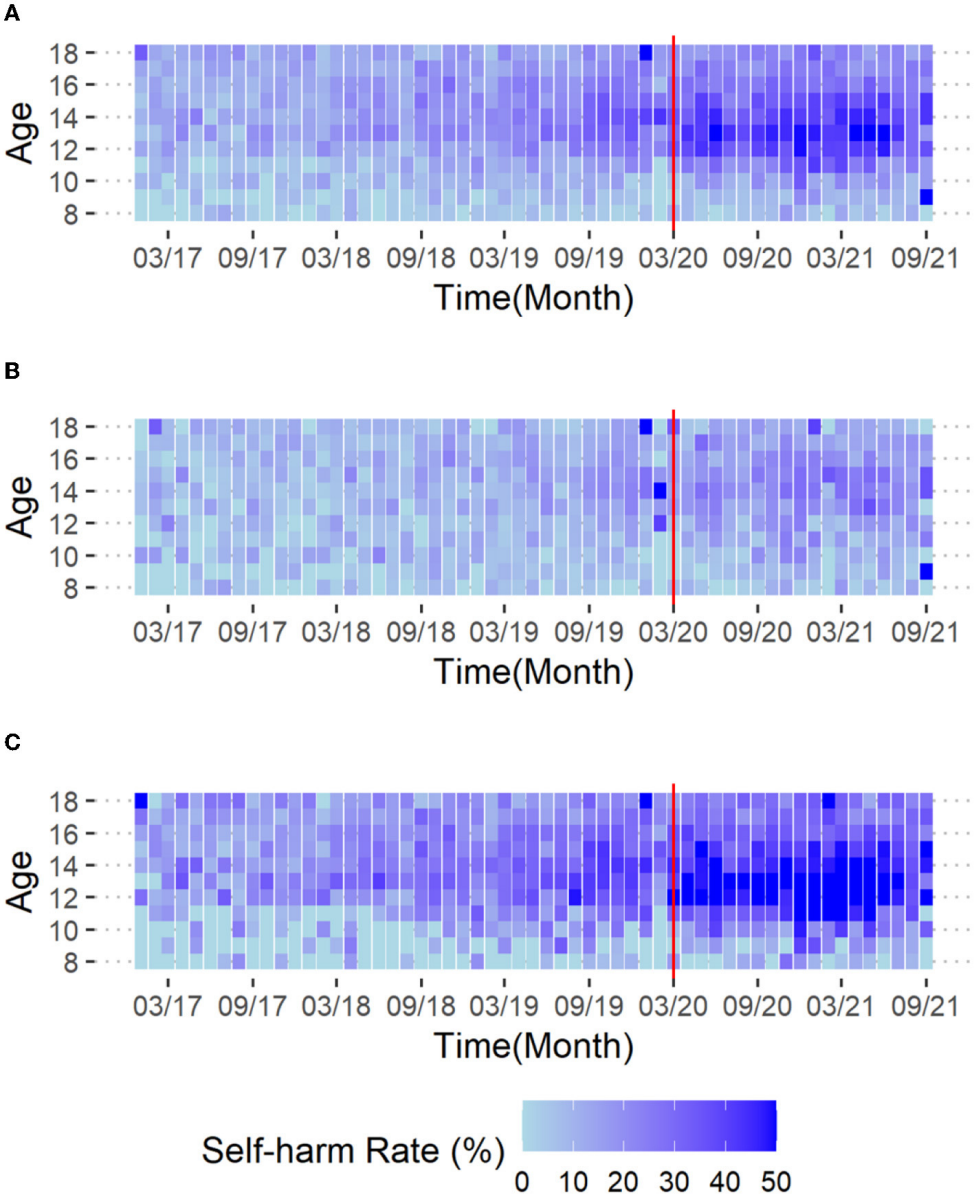
Heat map of self-harm rates. **(A)** Entire sample, **(B)** male, and **(C)** female patients. The colors in the cells represent the self-harm rate for each age group in each month. The red vertical line indicates March 2020, from which time the adolescent's social environment was affected (nation-wide home study started).

Visualization of the monthly data further suggests an association between the COVID-19 society-wide isolation period (start from March 2020) and changes in self-harm rates (Figure 2). The overall self-harm rate among adolescents aged 11 to 16 years increased after March 2020 (Figure 2A). This trend was more pronounced among females (Figure 2C).

**Figure 3:**
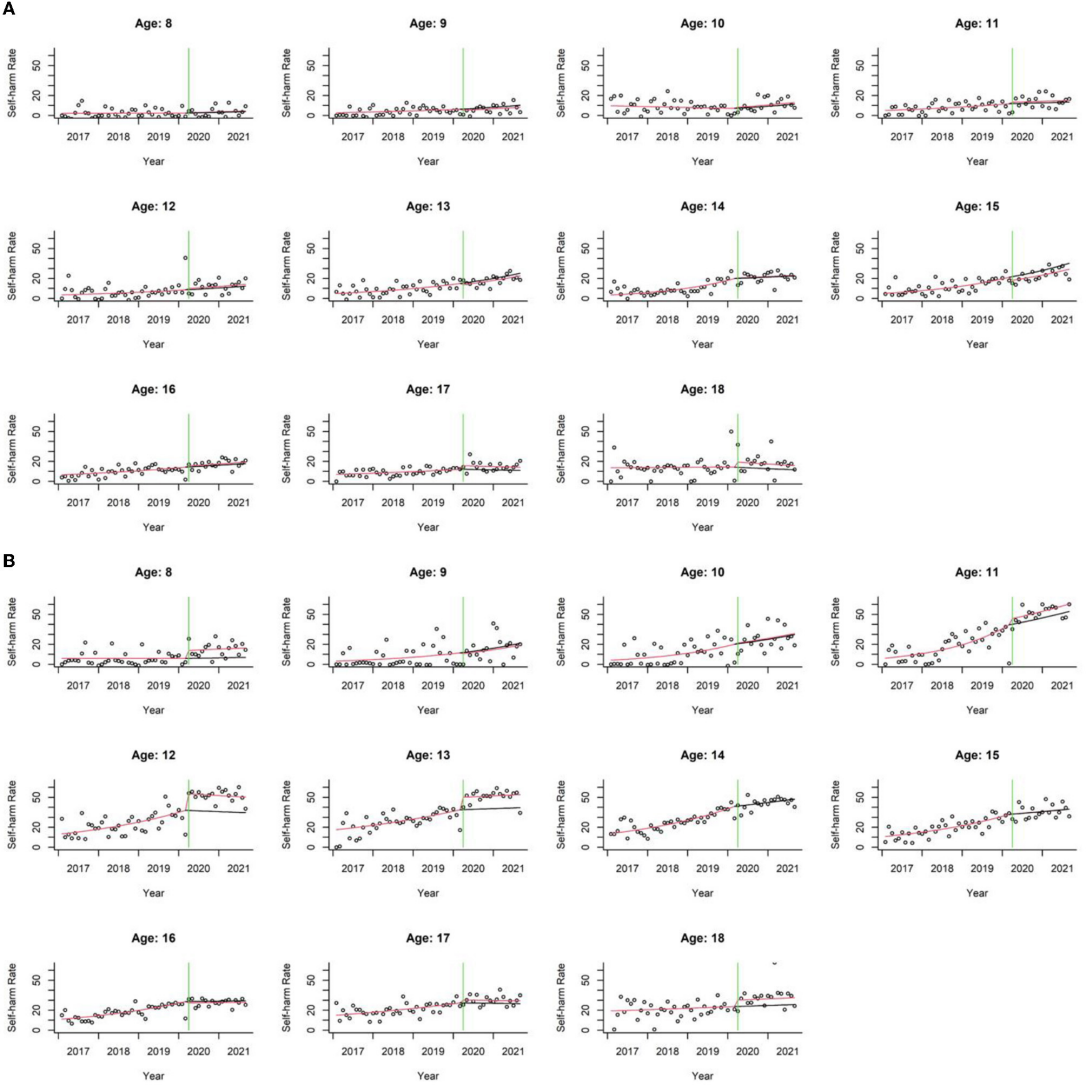
Scatter plots of self-harm rates over time for male **(A)** and female patients **(B)** in each age group. Seasonality was removed from the observed and predicted data (see Supplementary Figure S1 for the scatter plots of data containing the seasonality). The dots represent the observed monthly self-harm rates. The red line represents the predicted self-harm rate according to the ITS model, while the black line represents the predicted self-harm rate if COVID-19 society-wide isolation (starting from the green line) did not occur.

The ITS quantitatively disentangled the effect of COVID-19 society-wide isolation on self-harm rates from seasonal and global temporal trends. We found a clear global trend of increasing self-harm rates over time among males aged 13–17 years and females aged 10–17 years (Supplementary Table S2, *p*_fdr_ < 0.05). More importantly, after adjusting for global and seasonal temporal trends, COVID-19-related society-wide isolation significantly increased self-harm rates at specific ages and sexes (Figure 3; Supplementary Figure S1). Specifically, the society-wide isolation showed no significant effect in males, but significantly elevated self-harm rates in female patients at age 12 [RR 1.45 (95% CI 1.19–1.77); *p*_fdr_ = 0.0031] and 13 [RR 1.33 (95% CI 1.15–1.5); *p*_fdr_ = 0.0031, Supplementary Table S3]. In addition, the “slope” of the self-harm rate, representing alterations of the COVID-19 effect after March 2020, was significantly negative for females aged 12–16 years (*p* < 0.05, Supplementary Table S4). Combined with the “COVID-19” effect, the results suggest an overall increase in the self-harm rate after March 2020, but with a trend toward a slower increase in females aged 12–16 years. No significant slope change was found for male patients.

**Figure 4:**
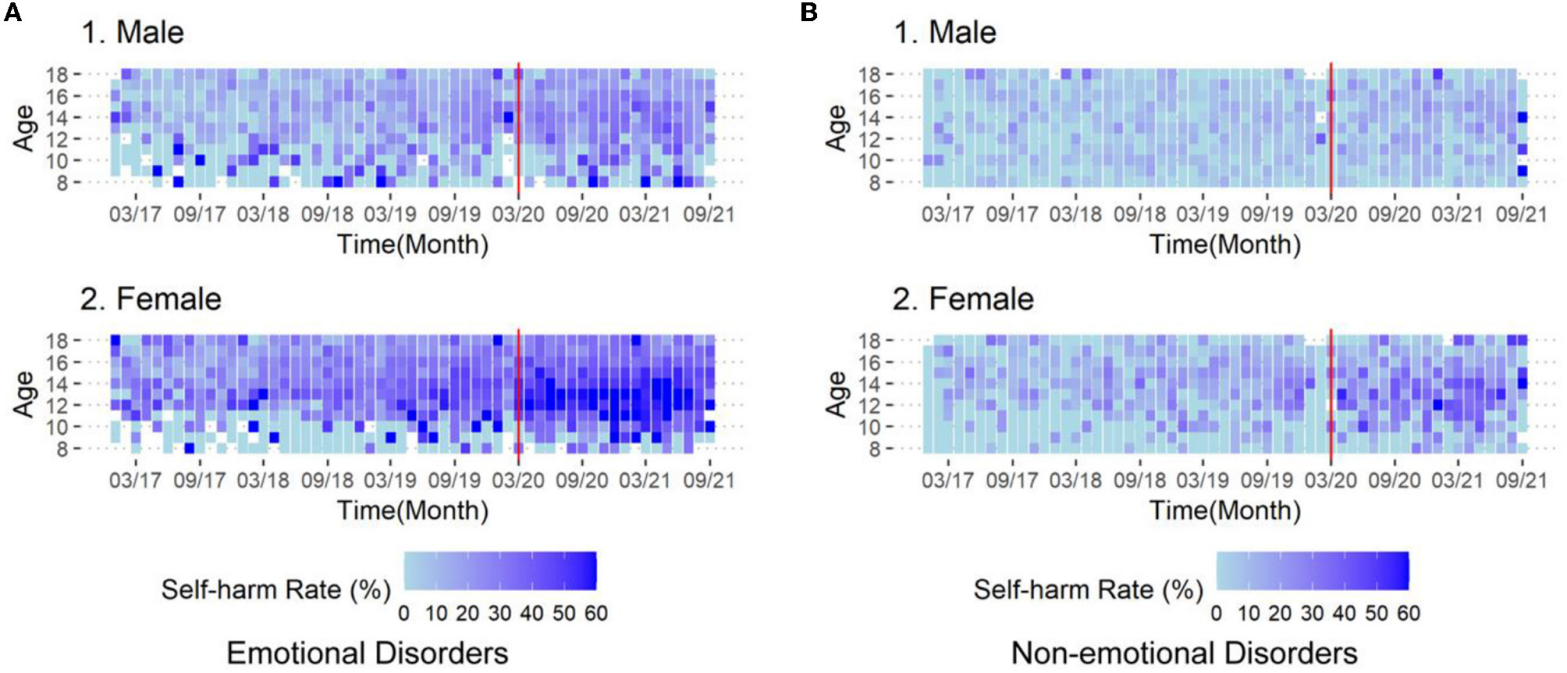
Heat map of self-harm rates for (1) male and (2) female patients with **(A)** emotional disorders and **(B)** non-emotional disorders. The colors in the cells represent the self-harm rate for each age group in each month. The red vertical line indicates March 2020, from which time the adolescent's social environment was affected (national-wide home study started). Emotional disorders include depressive and bipolar disorder, anxiety disorder, post-traumatic stress disorder, obsessive compulsive disorder and childhood emotional disorder.

Compared to other mental disorders, we found more pronounced increases in self-harm rates in females with emotional disorders (Figure 4). In the ITS analysis, we focused on patients (with psychiatric diagnoses) aged 11–13 because of their significant COVID-19-related changes (Figure 3). Females with emotional disorders at 12 years [RR 1.37 (95% CI 1.1–1.7); *p*_fdr_ = 0.041**]** and 13 years [RR 1.31 (95% CI 1.11–1.53); *p*_fdr_ = 0.021] were significantly affected by the COVID-19 society-wide isolation (Supplementary Figures S2–S3; Supplementary Table S5). In contrast, this phenomenon was not significant in females with other mental disorders and all males.

## 4. Discussion

By modeling large-scale medical record data, we found that 10–17 year-old females and 13–16 year-old males with mental health problems showed alarmingly elevated self-harm rates in recent years. Further, after adjusting for global and seasonal variation in self-harm rates, we confirmed that the COVID-19-related society-wide isolation significantly elevated self-harm rates in females aged 12–13 years. Furthermore, for the first time, this study shows the peak age for the prevalence of self-harm among children and adolescents with mental health problems in East China, suggesting that it is earlier than the high prevalence age of 15–17 years among general population reported in the literature of other countries (38, 39). This study alerts clinicians to the need for concern about the risk of self-harm in early adolescents with mental health problems in clinical practice.

One of our key findings is that the society-wide isolation due to COVID-19 selectively increases the risk of self-harm in female patients aged 12–13 years. Previous studies have reported an increase in the rate of health care visits for self-harm in adolescent populations during the COVID-19 pandemic, such as the UK population aged 10–17 years (21), a 10-country European and West Asian population under 18 years of age (29), an Australian population aged 12–17 years (20, 40), and a Chinese psychiatric inpatient sample under 18 years of age (41). The current study differs from the previous studies in revealing the age-dependence of the effects of COVID-19-related prevention measures, adjusting for overall temporal trends and seasonal variations. The fine-grained controls for confounding variables support the solidity of the results. The precise year-to-year analyses revealed environmental impacts and age-specific characteristics of self-harming behaviors among adolescents with mental health problems in East China. The findings suggest that self-harming behaviors of females aged 12–13 years are sensitive to the society-wide isolation.

Beyond changes in social relationships, multiple factors attributed to the society-wide isolation can explain the increase of self-harm in early adolescents in China. First, the quarantine and home study measures have increased early adolescents' exposure to electronic devices (42), cyberbullying (43), and parent-child conflict (44), but led to decreased peer interaction. These environmental changes exacerbated anxiety and depression in children and adolescents. This pathway is a dominant cause for self-harming behaviors (45). Furthermore, in China, ages 11–13 are the transition period from elementary to middle school, when students need to compete academically to gain admission to more advanced schools. Students only have one chance to choose a better school, so there is considerable stress on students and families (46). Coupled with school closures during the pandemic, students may experience a decline in academic achievement and thus experience increased psychological stress (47). Therefore, the biological vulnerability combined with the stressors associated with COVID-19 may contribute to a greater risk of self-harm behaviors in early adolescence (48).

Another insight of the findings is that COVID-19-related society-wide isolation has increased the risk of self-harm among adolescents toward a younger age. Our data showed a 37.3–44.78% detection rate of self-harming behaviors among 11-year-old female children after the onset of pandemic-associated society-wide isolation. This value exceeded the peak level across all ages before the pandemic (36.38%), suggesting the need to focus on the occurrence and factors influencing self-harm behaviors from a younger age. These findings are in contrast with a recent paper that reported negative findings on the associations between excess of self-harm requiring health care and the COVID-19 pandemic (19). This difference can be explained by the fact that the two studies sampled populations with different levels of severity of self-harming behaviors. While the present study included all self-harming behaviors that could be of concern to psychiatrists and documented in the medical record, the Ray et al. study focused on self-harming behaviors that were severe enough to require emergency department management. Thus, self-harm in children and adolescents may require more attention from the mental health field.

Multiple factors help explain the sex dependence of self-harm rates. First, females are more likely to adopt emotion-focused coping strategies in early adolescence (49). In this context, self-harm can be seen as a negative strategy or symptoms of adolescents' coping method with stress or suffering (12). Second, sex differences in brain developmental processes (50) and hormonal changes related to neural activity (51) also make females more vulnerable in early adolescence to emotional distress and in need of peer feedback and companionship (52). Social isolation is more likely to lead to abnormal emotional experiences and depressive symptoms (53). In addition, some studies have found that female adolescents are more susceptible to the impact of the self-harming behaviors of their peers (54).

In addition, our study found that the rate of self-harm was significantly higher after the pandemic in patients with diagnoses of emotional disorders compared with those with other mental disorders (including developmental disorders, schizophrenia, and other behavioral problems). To our knowledge, this is the first report of the differences in the detection rate of self-harm among patients with different mental disorders in the pandemic. This finding calls for more attention to self-harm behaviors in adolescents with emotional disorders.

This study has some limitations. First, the sample was from a large mental health center in Shanghai. Due to divergence in socioeconomic development and personal preferences, the population is not representative of the less developed or rural areas in China. Second, medical records usually present a single-question inquiry for self-harming behaviors, and the detection rate of this approach was generally lower than that of the scale findings, because self-harm behavior information received from patients and caregivers was within a limited time and may have been neglected or denied (55). Therefore, potential measurement error needs to be considered when using the specific values. Third, the retrospective nature of the data may be an additional source of error.

## 5. Conclusion

The prevalence of self-harm among children and adolescents with mental health problems in East China has alarmingly increased in the past 5 years and exhibits remarkable age- and sex-dependence. The society-wide isolation due to COVID-19 selectively increased the risk of self-harm among female adolescents in early adolescents, especially in those with emotional disorders. Prevention and early identification and intervention may need to move forward from mid-adolescence to early adolescence, with particular attention to females with mental health problems in early adolescence.

## Data availability statement

The original contributions presented in the study are included in the article/Supplementary material, further inquiries can be directed to the corresponding authors.

## Ethics statement

The studies involving human participants were reviewed and approved by Shanghai Mental Health Center. Written informed consent was not required by the participants' legal guardian/next of kin.

## Author contributions

WL and ZH wrote the initial analysis plan, conducted the analysis, and produced figures. WL, ZH, ZY, and WC wrote the first draft of the manuscript. All authors conceptualized the study and contributed to its design and contributed to editing and commenting on the final version. All authors contributed to the article and approved the submitted version.
