# Supplementary material for: Age- and sex-dependent increase in self-harm among adolescents with mental health problems in East China during COVID-19 related society-wide isolation
Source: Front Public Health. 2023 Mar 9;11:1129123. doi: 10.3389/fpubh.2023.1129123 (PMC10036048; doi:10.3389/fpubh.2023.1129123)
Supplement: Supplementary file 1 [file Table_1.pdf]

Supplementary Materials for

Age- and sex-dependent increase in self-harm among adolescents with mental health problems in East China during COVID-19 related society-wide isolation

Wenjing Liu[1,#], Zhishan Hu[2, 3,#], Zhen Liu[1], Fang Zhang[4], Yue Ding[2, 3],Ying Shui[1], Zhi Yang[2, 3]* , Wenhong Cheng[1, 4]*

1. Department of Child and Adolescent Psychiatry, Shanghai Mental Health Center, Shanghai Jiao Tong University School of Medicine, Shanghai, China

2. Laboratory of Psychological Heath and Imaging, Shanghai Mental Health Center, Shanghai Jiao Tong University School of Medicine, Shanghai, China

3. Institute of Psychological and Behavioral Science, Shanghai Jiao Tong University, Shanghai, China

4. Department of Psychological Medicine, Shanghai General Hospital, Shanghai Jiao Tong University School of Medicine, Shanghai, China

# Equal contribution

Corresponding authors:

*Zhi Yang; E-mail: yangz@smhc.org.cn

*Wenhong Cheng; E-mail: chengwhb@aliyun.com

Supplementary tables

Table S1 Chinese terms used in the search to express self-harming behavior and their corresponding English terms

English term indicating self-harm behaviors	Corresponding Chinese terms used in the search
Ottawa self-injury inventory	
Cutting	划伤/划自己/划伤自己/划腿/划伤腿/划伤大腿/划伤小腿/划胳膊/划伤胳膊/划上臂/划伤上臂/划手腕/划伤手腕/划手背/划伤手背/划脸/划伤脸
Scratching	搔抓/抓伤/抓伤自己
Interfering with wound healing	揭掉伤疤/撕伤疤/防止伤口愈合/妨碍伤口愈合
Burning	烫伤/烫自己/烧伤/烧自己
Biting	啃咬/咬自己
Hitting	击打/打自己/撞墙/撞自己/撞伤/
Hair pulling	拔头发
Piercing skin with sharp pointy objects	刺伤/刺伤自己
Piercing of body parts	刺伤/刺伤自己
Headbanging	撞头
Other (specify):	抠皮(picking skin)/ 吞药 (self-harm with drugs)/ 自伤行为, 自伤, 自残, 弄伤 (all means "self-harm")
Suicide Attempts	
Jump from building	跳楼

Hanging	上吊/自缢
Slit wrist to commit suicide	割腕自杀
Suicide attempts	自杀未遂/消极行为
**Self-harm behavior or suicide attempts checked in system**	
Self-harm	有自伤行为/自伤
Suicide attempts	自杀/消极行为

Note: We did not include those who drink or eat anything to commit self-harm.

Table S2 Effect of global temporal trend on self-harm rate

Sex	Age	β	SE	t value	p	p_{fdr}
Male	8	0.003	0.02	0.16	0.87	0.87
	9	0.02	0.01	1.77	0.084	0.12
	10	-0.01	0.01	-0.76	0.45	0.55
	11	0.02	0.01	1.96	0.056	0.090
	12	0.02	0.01	1.95	0.057	0.090
	13	0.03	0.01	3.66	0.0006	0.0023
	14	0.05	0.01	5.38	<0.0001	<0.0001
	15	0.04	0.01	5.43	<0.0001	<0.0001
	16	0.02	0.01	3.36	0.0015	0.0042
	17	0.02	0.01	2.55	0.014	0.031
	18	0.001	0.01	0.18	0.85	0.87
Female	8	0.0002	0.03	0.01	0.99	0.99
	9	0.03	0.03	1.30	0.20	0.25
	10	0.04	0.02	2.42	0.019	0.027

11	0.05	0.01	5.41	<0.0001	<0.0001
12	0.03	0.01	5.43	<0.0001	<0.0001
13	0.02	0.004	5.42	<0.0001	<0.0001
14	0.03	0.003	8.65	<0.0001	<0.0001
15	0.03	0.004	6.71	<0.0001	<0.0001
16	0.03	0.003	8.63	<0.0001	<0.0001
17	0.02	0.004	3.68	0.00058	0.00091
18	0.01	0.01	0.94	0.35	0.39

Note: β, estimation of the global trends; SE, standard error; $\boldsymbol{p_{fdr}}$, significance after FDR correction.

Table S3 Effect of COVID-19 related social isolation on self-harm rate

Sex	Age	RR (95% CI)	β	SE	t value	p	p_{fdr}
	8	1.14 (0.37, 3.51)	0.13	0.57	0.22	0.82	0.91
	9	0.81 (0.37, 1.8)	-0.21	0.41	-0.51	0.61	0.91
	10	1.14 (0.5, 2.62)	0.14	0.42	0.32	0.75	0.91
	11	1.12 (0.59, 2.14)	0.12	0.33	0.35	0.73	0.91
	12	1.15 (0.62, 2.14)	0.14	0.32	0.43	0.67	0.91
Male	13	0.9 (0.6, 1.36)	-0.11	0.21	-0.51	0.61	0.91
	14	1.02 (0.7, 1.49)	0.02	0.19	0.09	0.93	0.93
	15	0.83 (0.59, 1.18)	-0.18	0.18	-1.04	0.31	0.91
	16	1.08 (0.77, 1.52)	0.08	0.17	0.43	0.67	0.91
	17	1.29 (0.91, 1.83)	0.25	0.18	1.44	0.16	0.91

	18	1.37 (0.82, 2.27)	0.31	0.26	1.20	0.23	0.91
	8	2.37 (0.6, 9.34)	0.86	0.70	1.24	0.22	0.61
	9	0.91 (0.24, 3.47)	-0.10	0.68	-0.14	0.89	0.99
	10	1.04 (0.49, 2.22)	0.04	0.39	0.10	0.92	0.99
	11	1.14 (0.8, 1.63)	0.13	0.18	0.73	0.47	0.83
	12	1.45 (1.19, 1.77)	0.37	0.10	3.70	0.00056	0.0031
Female	13	1.33 (1.15, 1.55)	0.29	0.08	3.72	0.00052	0.0031
	14	1 (0.87, 1.15)	0.001	0.07	0.01	0.99	0.99
	15	1 (0.82, 1.23)	0.003	0.10	0.03	0.97	0.99
	16	0.95 (0.81, 1.11)	-0.05	0.08	-0.63	0.53	0.83
	17	1.11 (0.86, 1.42)	0.10	0.13	0.80	0.43	0.83
	18	1.27 (0.9, 1.79)	0.24	0.18	1.35	0.18	0.61

Note: RR, rate ratio; β, estimation of the COVID-19 social isolation effect; SE, standard error; $\boldsymbol{p_{fdr}}$, significance after FDR correction.

Table S4 Effect of 'slope' reflecting changes in self-harm rates with COVID-19 control measures

Sex	Age	β	SE	t value	p	p_{fdr}
	8	0.03	0.04	0.60	0.55	0.78
	9	0.01	0.03	0.17	0.87	0.96
Male	10	0.04	0.03	1.10	0.28	0.78
	11	-0.02	0.03	-0.58	0.56	0.78

	12	-0.004	0.03	-0.15	0.88	0.96
	13	-0.001	0.02	-0.05	0.96	0.96
	14	-0.04	0.02	-2.46	0.017	0.19
	15	-0.01	0.01	-0.68	0.50	0.78
	16	-0.01	0.01	-0.57	0.57	0.78
	17	-0.02	0.01	-1.48	0.15	0.78
	18	-0.01	0.02	-0.58	0.56	0.78
Female	8	0.01	0.06	0.17	0.87	1.00
	9	-0.0001	0.05	-0.002	1.00	1.00
	10	-0.02	0.03	-0.70	0.49	0.67
	11	-0.03	0.02	-2.22	0.031	0.057
	12	-0.03	0.01	-3.77	0.00045	0.0025
	13	-0.02	0.01	-2.77	0.0080	0.022
	14	-0.02	0.01	-3.39	0.0014	0.0052
	15	-0.02	0.01	-2.47	0.017	0.038
	16	-0.02	0.01	-3.76	0.00046	0.0025
	17	-0.02	0.01	-1.69	0.10	0.15
	18	-0.001	0.02	-0.07	0.95	1.00

Note: β, estimation of the global trends; SE, standard error; p_{fdr}, significance after FDR correction.

Table S5 Effects of social isolation associated with COVID-19, stratified by type of mental disorder.

Gender	Disorder	Age	RR (95% CI)	β	SE	t value	p	p_{fdr}
Male	Emotion	11	0.52 (0.17, 1.57)	-0.66	0.57	-1.16	0.25	0.77
		12	1.1 (0.56, 2.15)	0.09	0.34	0.27	0.79	0.99
		13	0.82 (0.57, 1.17)	-0.20	0.19	-1.09	0.28	0.77
	Other	11	2.04 (0.9, 4.63)	0.71	0.42	1.71	0.094	0.52
		12	0.71 (0.22, 2.31)	-0.34	0.60	-0.57	0.57	0.79
		13	0.72 (0.27, 1.92)	-0.33	0.50	-0.65	0.52	0.79
Female	Emotion	11	1.02 (0.69, 1.51)	0.02	0.20	0.09	0.93	0.95
		12	1.37 (1.1, 1.7)	0.31	0.11	2.80	0.0074	0.041
		13	1.31 (1.11, 1.53)	0.27	0.08	3.28	0.0019	0.021
	Other	11	1.1 (0.37, 3.25)	0.09	0.55	0.16	0.87	0.91
		12	1.45 (0.73, 2.87)	0.37	0.35	1.07	0.29	0.91
		13	1.54 (0.85, 2.79)	0.43	0.30	1.42	0.16	0.91

Note: RR, rate ratio; β, estimation of the COVID-19 social isolation effect; SE, standard error; $\boldsymbol{p_{fdr}}$significance after FDR correction. Patients with emotional disorders including *depressive and bipolar disorder, anxiety disorder, post-traumatic stress disorder, obsessive compulsive disorder and childhood emotional disorder*.

Supplementary figures

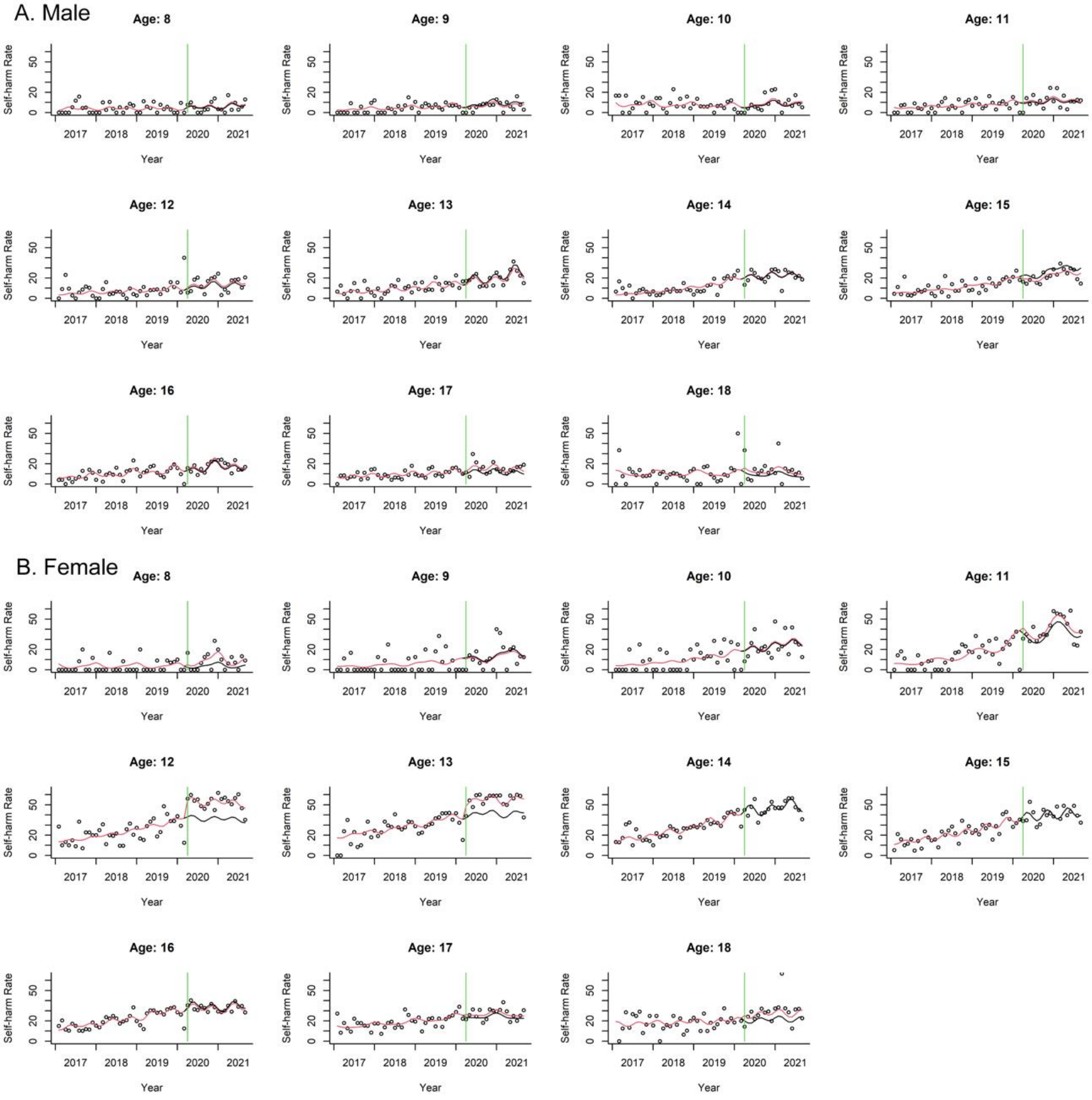

Figure S1 Scatter plot of self-harm rate of male (A) and female (B) patients over time. The red lines represent the predicted self-harm rate according to the ITS model. Black lines represent the predicted self-harm rate if the outbreak of COVID-19 did not happen.

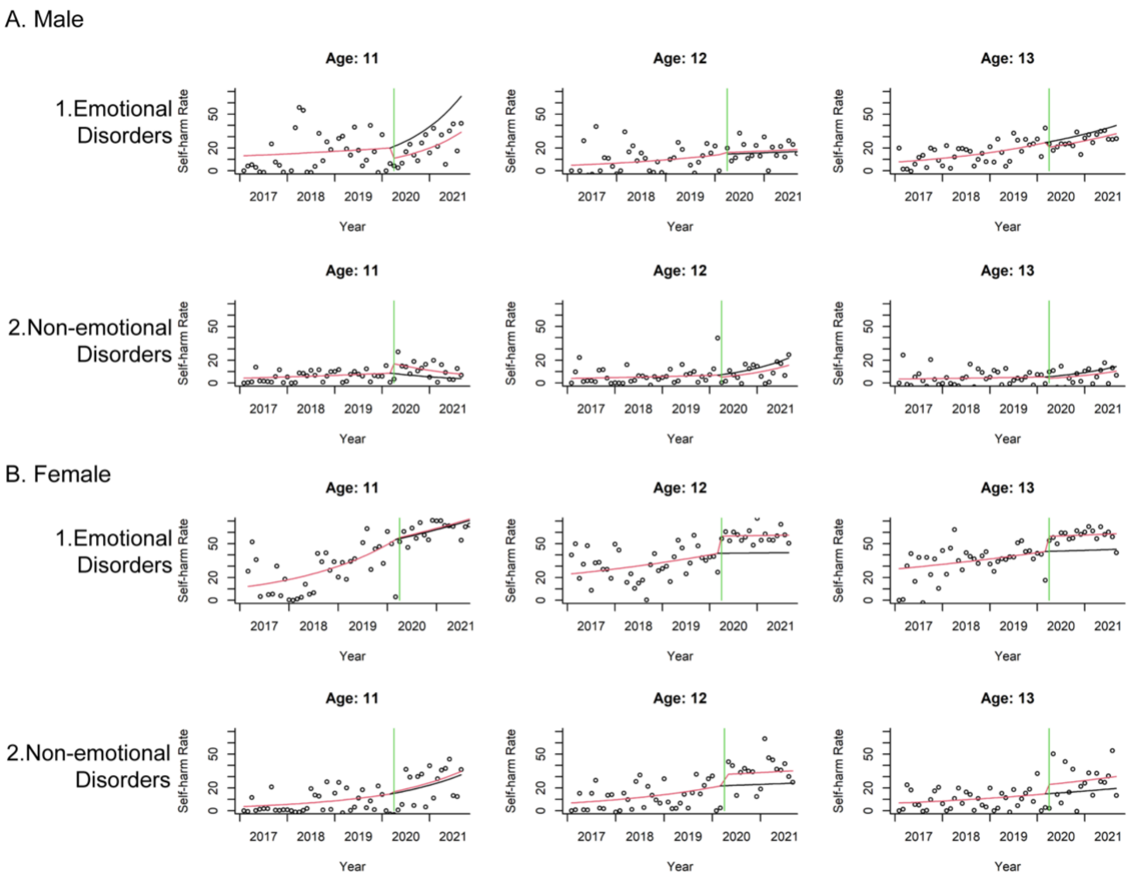

Figure S2 Scatter plots of self-harm rates over time for male (A) and female (B) patients with (1) emotional and (2) non-emotional psychiatric disorders. Seasonality was removed from the observed and predicted data. The dots represent the observed monthly self-harm rates. The red line represents the predicted self-harm rate according to the ITS model, while the black line represents the predicted self-harm rate if COVID-19 social isolation (starting from the green line) did not occur.

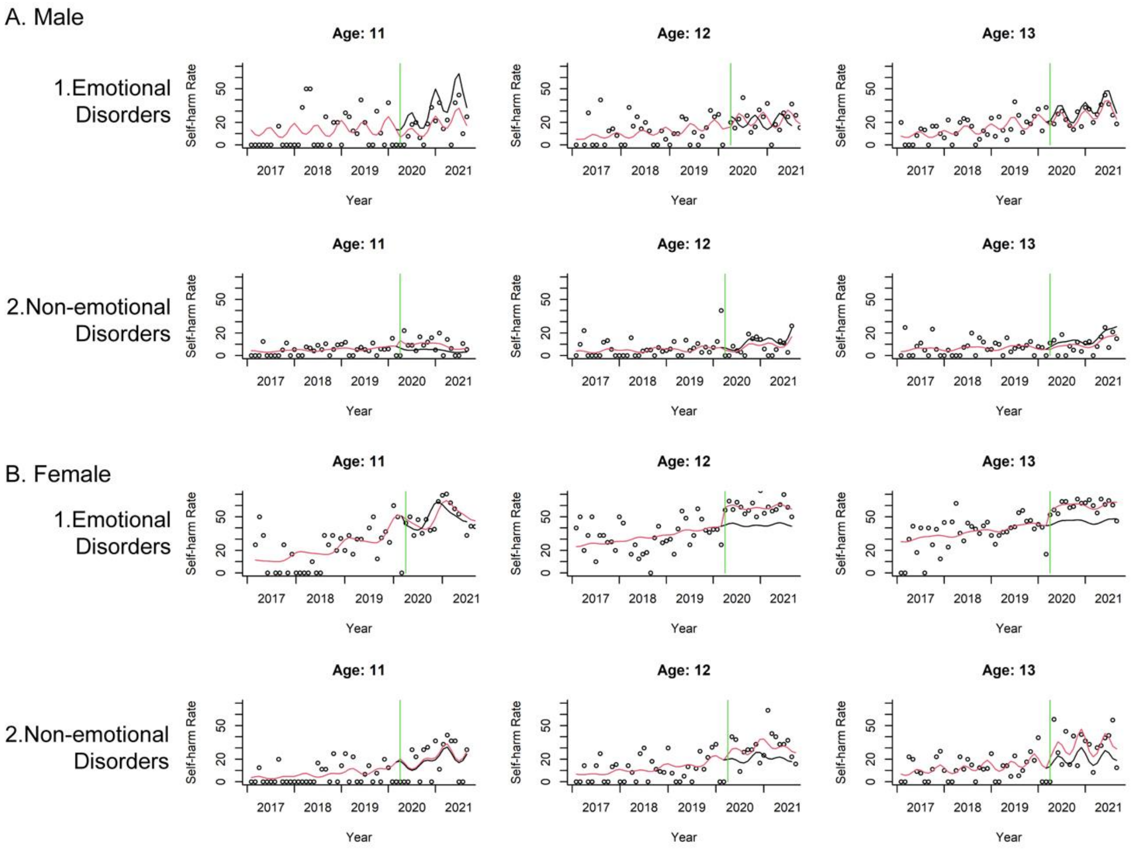

Figure S3 Scatter plot of self-harm rate of male (A) and female (B) patients over time regarding (1) emotional and (2) other mental disorders. The red lines represent the predicted self-harm rate according to the ITS model. Black lines represent the predicted self-harm rate if the outbreak of COVID-19 did not happen.
